# Gene Co-occurrence Networks Reflect Bacteriophage Ecology and Evolution

**DOI:** 10.1128/mBio.01870-17

**Published:** 2018-03-20

**Authors:** Jason W. Shapiro, Catherine Putonti

**Affiliations:** a Department of Biology, Loyola University of Chicago, Chicago, Illinois, USA; b Department of Computer Science, Loyola University of Chicago, Chicago, Illinois, USA; c Bioinformatics Program, Loyola University of Chicago, Chicago, Illinois, USA; d Department of Microbiology and Immunology, Loyola University of Chicago, Maywood, Illinois, USA; Northern Arizona University

**Keywords:** bacteriophage evolution, bacteriophages, networks, virus host range

## Abstract

Bacteriophages are the most abundant and diverse biological entities on the planet, and new phage genomes are being discovered at a rapid pace. As more phage genomes are published, new methods are needed for placing these genomes in an ecological and evolutionary context. Phages are difficult to study by phylogenetic methods, because they exchange genes regularly, and no single gene is conserved across all phages. Here, we demonstrate how gene-level networks can provide a high-resolution view of phage genetic diversity and offer a novel perspective on virus ecology. We focus our analyses on virus host range and show how network topology corresponds to host relatedness, how to find groups of genes with the strongest host-specific signatures, and how this perspective can complement phage host prediction tools. We discuss extensions of gene network analysis to predicting the emergence of phages on new hosts, as well as applications to features of phage biology beyond host range.

## IMPORTANCE

Bacteriophages (phages) are viruses that infect bacteria, and they are critical drivers of bacterial evolution and community structure. It is generally difficult to study phages by using tree-based methods, because gene exchange is common, and no single gene is shared among all phages. Instead, networks offer a means to compare phages while placing them in a broader ecological and evolutionary context. In this work, we build a network that summarizes gene sharing across phages and test how a key constraint on phage ecology, host range, corresponds to the structure of the network. We find that the network reflects the relatedness among phage hosts, and phages with genes that are closer in the network are likelier to infect similar hosts. This approach can also be used to identify genes that affect host range, and we discuss possible extensions to analyze other aspects of viral ecology.

## INTRODUCTION

Bacteriophages (phages) are viruses that infect bacteria, and with over 10^31^ estimated on the planet, are often the most abundant and diverse members of any ecosystem (1). Phages act as predators, drivers of biogeochemical cycles (2), industrial contaminants (3), and important mutualists within bacterial pathogens that cause disease in plants and animals (4, 5). Phages have also been used as therapeutics in agriculture (6) and for treating antibiotic-resistant bacterial infections (7, 8).

There are no universal genes shared by all phages, and horizontal gene transfer (HGT) between viruses is common (9). In essence, every phage genome is a mosaic that reflects the often disparate evolutionary histories of its genes (9, 10), and traditional phylogenetic methods can only be applied at relatively narrow levels of diversity where signature genes are shared among the genomes under consideration. It is therefore difficult to place phage taxa in a broader evolutionary context (though see reference 11 for an example based on protein folding). To overcome these challenges, network-based approaches have been used to depict the relationship between phage genomes on the basis of the similarity of their genic content or overall sequence identity (12–16). Bipartite networks have also been made to show the links between genes and genomes (17).

Genome-level network analyses are useful, because they make it possible to visualize distant phage relationships in place of phylogenies (12, 16). These approaches have also shown how modules of genes underlying the genome network may relate to different phage lifestyles and potentially host range (16, 18). At the same time, genome-level networks take the focus away from the targets of selection: genes. In the present work, we build a network of genes where genes are connected if they are ever found within the same genome. By focusing on the gene level, it is possible to address new questions in virus ecology and evolution.

Host range, in particular, constrains viral ecology and evolution and is expected to play a critical role in shaping patterns of gene exchange among viruses. Host range typically depends on individual virus-host gene interactions (19), and both phages and their hosts can acquire genes that alter these interactions through HGT (20–22). In eukaryotic interactions, comparative phylogenetics is often used to test whether hosts and their pathogens have codiverged (23). Similar approaches have also been applied at the strain level to show how eukaryotic viruses have evolved and changed hosts over time (24). While these methods can also be applied to phage evolution (e.g., see reference 25), their value evaporates when considering diverse phages that may have no genes in common.

Here, we build a gene-level network representing the co-occurrence of genes across phage genomes. This network provides a robust view of virus genetic diversity and a basis for placing clusters of genes in an ecological and evolutionary context. The network topology also reflects the evolutionary relationships among phage hosts: genes from phages infecting related hosts group together in the network according to the phylogenetic relatedness of their hosts. Further, we developed an algorithm to identify genes whose presence or absence has the strongest correspondence to phage host range, and we demonstrate how it may be used to complement existing host prediction methods.

### Building genome and gene level networks.

**FIG 1:**
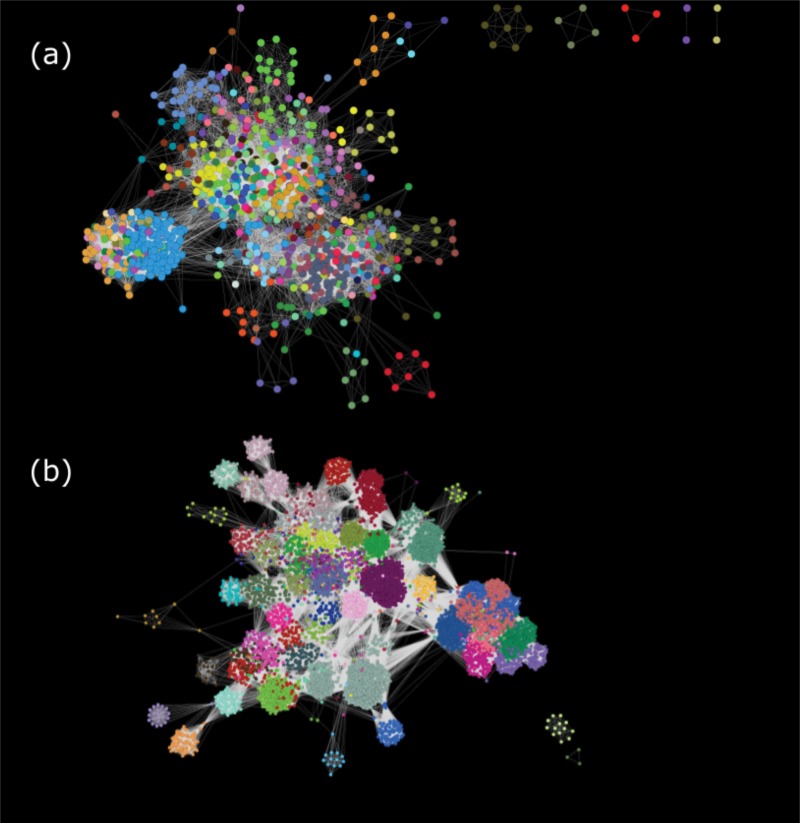
Genome (a) and gene (b) networks colored on the basis of their membership in graphical clusters identified using MCL with the inflation parameter set to 6 for the genome network and to 4.1 for the gene network.

We built genome- and gene-level networks for a set of 945 phage RefSeq genomes, consisting of 92,801 gene sequences. In the genome network (Fig. 1a), nodes represent virus genomes, and two nodes are connected if they share at least one gene. In the gene network (Fig. 1b), nodes represent homologous phage protein sequences, and two nodes are connected if these genes are ever found in the same genome. Homologous genes were identified with as low as 35% identity via clustering by USEARCH (26). Singleton and doubleton clusters were removed from consideration to increase the reliability of connections between genes. This filter yielded a final set of 8,847 gene clusters from across 913 phage genomes, excluding 32 phage genomes from primarily undersampled, tailless phage families.

In each network, there exist subsets of nodes that form subgraphs in which members have more connections in common with each other than with the rest of the network. We formally identified these subsets of interconnected nodes using the Markov Cluster algorithm (MCL) (27). MCL relies on an inflation parameter that transforms the adjacency matrix of the underlying network. Higher inflation values generally yield more clusters, and others have measured cohesion within subgraphs using the intracluster clustering coefficient (ICCC) to optimize this parameter choice (13, 16). Based on the ICCC, we chose inflation factors of 6 for the genome network and 4.1 for the gene network (see Fig. S1 in the supplemental material), corresponding to 209 and 135 clusters, respectively (shown as distinct colors in Fig. 1).

10.1128/mBio.01870-17.1 FIG S1  Scatterplot indicating how the ICCC varies with the MCL inflation parameter for both the genome network (red) and the gene network (black). Download FIG S1, EPS file, 0.1 MB. Copyright © 2018 Shapiro and Putonti. 2018 Shapiro and Putonti This content is distributed under the terms of the Creative Commons Attribution 4.0 International license.

### Clusters of phage genes are associated with known phage hosts.

**FIG 2:**
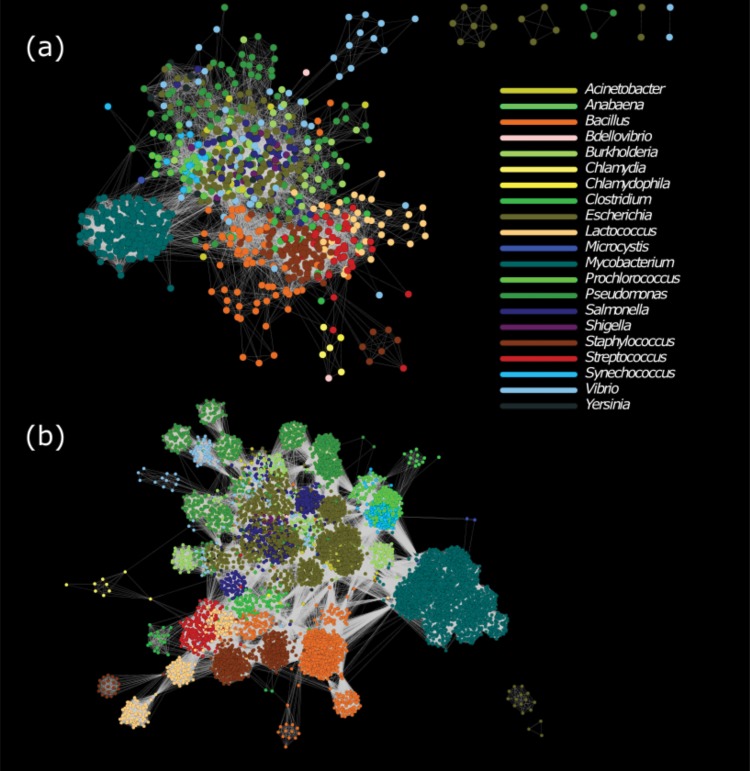
Genome (a) and gene (b) networks identical to those in Fig. 1a and b, respectively, except that the nodes have been colored to reflect the host genus associated with each phage. In the gene network, each node signifies a set of homologous sequences, and colors match the most common host for the genomes containing these homologs.

Given the gene and genome networks, we colored the nodes according to the phage host genus (Fig. 2). In the gene network, each node represents a set of homologous genes, and only the most common host associated with these homologs is indicated. Phage host appears to map more closely to graphical clusters in the gene network (Fig. 2b) than in the genome network (Fig. 2a).

The central mass of the gene network consists largely of genes from phages infecting enteric bacteria, primarily *Escherichia* and *Salmonella* but also *Vibrio*, *Yersinia*, *Acinetobacter*, and *Burkholderia*. More distinct subsets dot the periphery of the network and include several disparate clusters from *Pseudomonas*-infecting viruses, and a small set infecting cyanobacteria. These cyanophages are predominantly T4-like viruses with many genes in common with the T4-like phages infecting *Escherichia coli*.

The largest and most distinct cluster of phage genes corresponds to phages infecting *Mycobacterium smegmatis*, a nonpathogenic relative of *Mycobacterium tuberculosis*. These phages have been heavily sampled compared to other hosts because of the SEA-PHAGES program, in which undergraduates isolate and sequence phage genomes (28). Though phages of other *Mycobacterium* species have not been studied as thoroughly, phages infecting *Mycobacterium smegmatis* have been shown to infect other *Mycobacterium* species, and genes from phages infecting *M. tuberculosis* are also present within this subgraph (29, 30).

Phages infecting Gram-positive bacteria contain genes in neighboring clusters in a separate region of the network. This region includes phages infecting *Lactococcus*, *Streptococcus*, *Staphylococcus*, *Clostridium*, and *Bacillus*. Within this space, one graph cluster includes genes from phages infecting either *Lactococcus lactis* or *Streptococcus thermophilus*, two bacteria commonly used in dairy fermentations (31). In the case of *Bacillus* and *Streptococcus*, phage genes form distinct clusters even at the host species level (see Fig. S2).

10.1128/mBio.01870-17.2 FIG S2  Gene network identical to those in Fig. 1b and 2b but with nodes for *Bacillus*, *Streptococcus*, *Burkholderia*, and *Prochlorococcus* relabeled at the species level to show additional contrasts where visible. Download FIG S2, TIF file, 1.1 MB. Copyright © 2018 Shapiro and Putonti. 2018 Shapiro and Putonti This content is distributed under the terms of the Creative Commons Attribution 4.0 International license.

### Quantifying associations between graph clusters and phage hosts.

**FIG 3:**
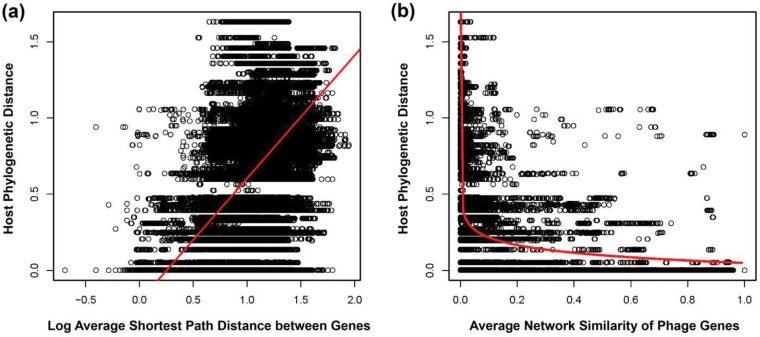
(a) Host phylogenetic distance (*y* axis) is positively correlated with the average shortest path distance between the genes in the gene network for pairs of phages. The plot and regression shown are based on log transformation of the average shortest path distance (*R*^2^ = 0.306, *P* < 10^−16^). (b) Genomes composed of genes with a greater proportion of shared edges in the network also infect more closely related hosts. The plot shown is untransformed, with regression from a log transformation of the node similarity measure (*R*^2^ = 0.3618, *P* < 10^−16^).

Visual inspection suggests that neighboring groups of genes in the network come from phages with the same or closely related hosts. To test this hypothesis, we first built a genus-level phylogeny (Fig. S3) of the phage hosts (see Materials and Methods). Because no single gene is shared among all phages, we could not build an analogous genus-level phylogeny for the phages. Instead, we summarized the relative positions of their genes in the gene network. To do so, we determined the shortest path between nodes in the gene network. We then iterated through each pair of phage genomes and calculated the average shortest path distance separating their respective genes. On average, the shortest path between any two genes in the network is 3.20 edges, and the average shortest path distance between two phage genomes is significantly positively correlated with the phylogenetic branch distance between their hosts (*R*^2^ = 0.306, *P* < 10^−16^; Fig. 3a).

10.1128/mBio.01870-17.3 FIG S3  Approximate maximum-likelihood tree of host genera infected by phages in the data set. Values along branches indicate the local bootstrap support for each split based on 1,000 resamples. Download FIG S3, EPS file, 0.1 MB. Copyright © 2018 Shapiro and Putonti. 2018 Shapiro and Putonti This content is distributed under the terms of the Creative Commons Attribution 4.0 International license.

We also estimated the proportion of shared edges between each pair of nodes in the gene network, sometimes referred to as node similarity. Similarity compares the topological position of each node in the network and should be more robust to the presence of highly connected nodes (or hubs) than the shortest path distance. Here, the average similarity of the genes in any two phage genomes is negatively correlated with the phylogenetic distance between phage hosts (*R*^2^ = 0.3618, *P* < 10^−16^; Fig. 3b) and explains more of the variation in host relatedness than the average shortest path distance. This means that phages whose genes share more edges in the gene network are more likely to infect the same host. This continuous relationship indicates that phages whose genes have intermediate topological similarity are more likely to infect hosts that are intermediately related.

### Accounting for host variation within nodes.

Many of the individual genes in the network are found in phages that infect different hosts, and this within-node diversity may provide insight into which genes affect host specificity. To account for this variation, we created a vector of host associations for each gene in the data set, enumerating how often each host genus is affiliated with the homologs found in other phage genomes. This host association vector provides a snapshot of how widespread each gene is among phages infecting different hosts. To capture the mosaicism within each genome, we then created a host association vector for each phage genome by summing across the host association vectors for each gene in the focal phage.

When summarizing the distribution of hosts in a gene or genome, it is important to account for both the relative frequency of each host and the fact that some hosts are more closely related than others. In community ecology, the mean pairwise phylogenetic distance (MPD) accomplishes these goals by weighting the average phylogenetic branch distance between taxa in a community by the relative abundance of each taxon (32). Here, we can think of each gene or genome as a community composed of associated hosts, and we calculate MPD by using the corresponding host association vector and the host genus tree.

**FIG 4:**
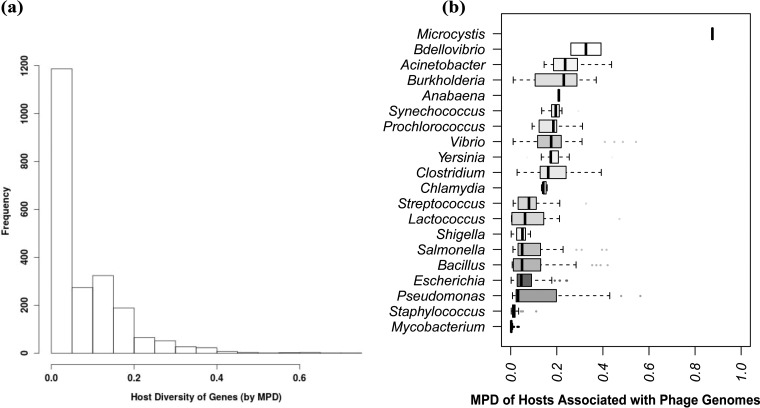
(a) Distribution of host association diversity, as measured by MPD, for each gene where at least two homologs are associated with two hosts (about 24.5% of the genes). Most such genes still display low host diversity. (b) Horizontal boxplot showing how MPD for whole phage genomes varies by the annotated host for each phage. Shading corresponds to relative sampling, with white representing <5 representative genomes and black representing >200.

Most individual genes have a relatively low MPD (Fig. 4a). In fact, 6,688 genes (75.5% of the data set) are associated with a single host genus. In contrast, only 132 of the 913 phage genomes consist entirely of genes affiliated with a single host. Moreover, the diversity of hosts associated with each phage genome varies with the annotated host of the phage. As shown in Fig. 4b, *Mycobacterium* and *Staphylococcus* phages contain genes that are almost exclusively associated with their respective hosts, whereas phages infecting other genera (e.g., *Burkholderia*) include genes that are found in phages infecting a wider variety of taxa. This variation mirrors the visible connections between nodes in the gene network, such as the broad dispersal of distinct clusters of genes from phages infecting *Pseudomonas*, *Vibrio*, and *Burkholderia*. MPD also accounts for the phylogenetic relatedness among *Escherichia*, *Salmonella*, and *Shigella* in reducing the diversity one might expect on the basis of the dispersal of genes infecting each in the middle of the network.

### Identifying genes that affect virus host range.

The topology of the gene network is influenced by the relatedness between phage hosts, but there is no reason to expect most virus genes to affect virus host range directly. Comparison of Fig. 1 and 2 shows that groups of genes that cluster together by the MCL algorithm are not always affiliated with the same host. To quantify the degree of correspondence between these alternative colorings of the gene network, we calculated the mutual information between MCL clusters and host associations.

Mutual information measures the extent to which knowing the state of one random variable can inform the state of additional variables. When applied to the gene network, the mutual information between MCL membership and host assignment is relatively low (1.42) because of the within-node variation in host association described in the previous section. In contrast, each node in the genome network can be assigned a single host on the basis of the GenBank annotation and doing so results in a higher mutual information value (2.18). MCL clusters in the gene network are also larger, on average (65.5 nodes), than clusters in the genome network (4.4 nodes) and, as noted above, may include many genes that do not affect host range. The latter effect also suggests that there may exist a subset of genes within the gene network that would provide greater correspondence to host associations.

To address this hypothesis, we developed an evolutionary algorithm, *mimax*, to identify the subset of genes that maximizes the mutual information of MCL clusters in the gene network and host associations. The *mimax* algorithm works as follows. In each iteration, a randomly selected MCL cluster in the gene network is removed from a matrix of cluster-host associations. If doing so would result in removing a phage genome from the data set, the deletion is rejected. If no genomes are lost, then the mutual information of the new matrix is calculated. If this value exceeds the value from the previous iteration, the deletion is retained; otherwise, it is rejected. Because *mimax* depends on the removal of uninformative clusters of genes, it should be more effective when there are more clusters from which to choose. When applied to the 135 MCL clusters previously found in the gene network, *mimax* removed 48 clusters containing 1,348 genes (~15% of the data set), resulting in a modest improvement in mutual information (to 1.57) but still falling short of the value observed in the genome network.

The most direct way to increase the granularity of MCL clusters is to increase the inflation parameter (see https://micans.org). Initially, we chose an inflation factor of 4.1 to optimize the ICCC, but this choice reduces the sensitivity of the *mimax* algorithm. Increasing the inflation factor to as high as 15 increases the number of MCL clusters to 513 (see Fig. S4). Adding random edges to the network can also increase the number of clusters found by MCL. The new edges create artificial distinctions between nodes that may otherwise have very similar sets of edges. When MCL explores the graph space, it will split larger clusters into smaller subsets. The number of clusters inferred increases monotonically with the number of additional edges (see Fig. S4), and the final mutual information following *mimax* is highest with five additional edges per node. Increasing inflation to 15 and adding 5 random edges per node yielded 1,355 MCL clusters.

10.1128/mBio.01870-17.4 FIG S4  Plot showing how the number of MCL clusters is affected by the inflation parameter (*x* axis) and adding random edges at different rates (colored circles, labeled). Download FIG S4, EPS file, 0.1 MB. Copyright © 2018 Shapiro and Putonti. 2018 Shapiro and Putonti This content is distributed under the terms of the Creative Commons Attribution 4.0 International license.

Given this new set of clusters, we ran *mimax* 10 times. In each replicate, the mutual information between MCL membership and host associations converged to a higher value (mean = 2.45) than that found in the genome network (see Fig. S5). On average, *mimax* reduced the number of MCL clusters and associated genes within the gene network to 483.5 and 4,070.6, respectively. These deletions suggest that the presence or absence of over half of the genes in the gene network is uninformative with respect to host range. Further, 72.5% of the retained genes were host-specific and 470.7 genomes consisted entirely of single-host genes. While *mimax* does not increase the host specificity of the individual genes retained (75.5% before *mimax*), it does increase the specificity of the genes in individual genomes (only 132 before *mimax*).

10.1128/mBio.01870-17.5 FIG S5  Mutual information increases monotonically with each iteration of *mimax* in all replicates. The red dashed line indicates the mutual information between clusters in the genome network and host association. Download FIG S5, EPS file, 0.3 MB. Copyright © 2018 Shapiro and Putonti. 2018 Shapiro and Putonti This content is distributed under the terms of the Creative Commons Attribution 4.0 International license.

**FIG 5:**
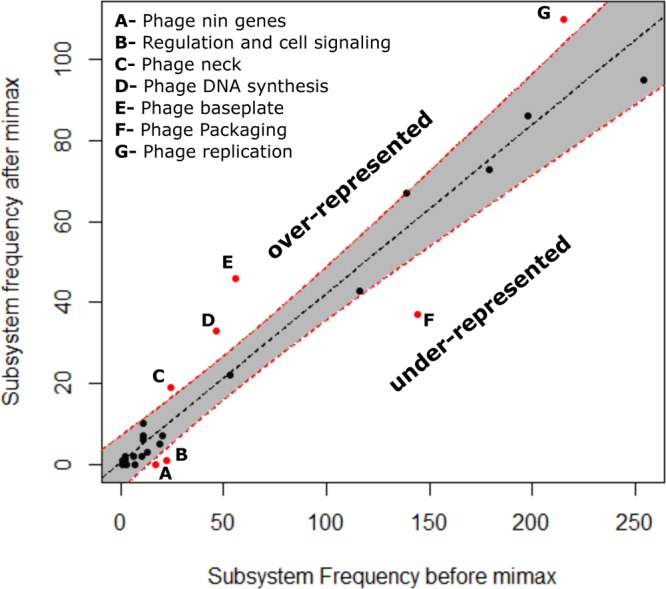
Plot showing a regression (*R*^2^ = 0.9292, *P* < 10^−16^) of the frequency of RAST-annotated subsystem functions (in Table 1) of genes after *mimax* in comparison to annotations before *mimax*. The dashed red lines and gray region outline the 99.9% confidence interval around the regression line. Red dots indicate subsystems that fall outside this confidence interval.

We also tested if the genes retained by *mimax* are associated with functions characteristic of phage-host interactions. We chose the *mimax* replicate with the highest mutual information and compared the frequency of non-hypothetical annotations of the remaining genes to the complete set by using RAST (33) (Table 1; Fig. 5). Phage baseplate, neck, replication, and DNA synthesis genes were significantly overrepresented following *mimax*, whereas phage packaging and bacterial regulatory genes were underrepresented. The overrepresented genes include functions known to affect host recognition and within-host phage reproduction (e.g., see reference 34), suggesting that gene function does affect *mimax* results.

**TABLE 1:** RAST annotations before and after *mimax*

Subsystem	Pre-*mimax*^a^	Post-*mimax*^a^	Proportion retained	99.9% CI^b^
Cofactors, vitamins, prosthetic groups, pigments	20	7	0.35	(2.948, 14.751)
Cell wall and capsule	11	6	0.5454545	(−1.105, 11.277)
Virulence, disease, defense	2	1	0.5	(−5.215, 7.862)
Photosynthesis	2	2	1	(−5.215, 7.862)
Miscellaneous	2	0	0	(−5.215, 7.862)
**Phage baseplate proteins**	**56**	**46**	**0.8214286**	**(18.421, 29.384)**
**Phage replication**	**215**	**110**	**0.5116279**	**(76.765, 104.007)**
**Phage packaging**	**144**	**37**	**0.2569444**	**(51.725, 69.671)**
**Phage neck proteins**	**24**	**19**	**0.7916667**	**(4.729, 16.316)**
Phage lysogenic conversion modules	7	0	0	(−2.925, 9.753)
Phage Ea cluster	10	2	0.2	(−1.559, 10.895)
Phage lysis modules	116	43	0.3706897	(41.582, 56.399)
**Phage DNA synthesis**	**46**	**33**	**0.7173913**	**(14.254, 25.189)**
IbrA and IbrB: coactivators of prophage gene expression	2	0	0	(−5.215, 7.862)
Phage tail proteins	254	95	0.3740157	(90.333, 123.054)
Phage virion particles involved in DNA ejection	11	10	0.9090909	(−1.105, 11.277)
Phage tail proteins 2	179	73	0.4078212	(64.148, 86.518)
**Phage nin genes−N-independent survival**	**17**	**0**	**0**	**(1.604, 13.586)**
Phage tail fiber proteins	139	67	0.4820144	(49.931, 67.284)
Phage capsid proteins	198	86	0.4343434	(70.821, 95.734)
Phage introns	19	5	0.2631579	(2.501, 14.362)
Membrane transport	2	1	0.5	(−5.215, 7.862)
Iron acquisition and metabolism	2	1	0.5	(−5.215, 7.862)
RNA metabolism	1	0	0	(−5.675, 7.486)
Nucleosides and nucleotides	53	22	0.4150943	(17.182, 28.115)
Protein metabolism	13	3	0.2307692	(−0.199, 12.044)
**Regulation and cell signaling**	**22**	**1**	**0.0454545**	**(3.84, 15.532)**
DNA metabolism	6	2	0.3333333	(−3.382, 9.373)
Respiration	1	1	1	(−5.675, 7.486)
Stress response	11	7	0.6363636	(−1.105, 11.277)
Amino acids and derivatives	3	0	0	(−4.756, 8.239)
Sulfur metabolism	1	0	0	(−5.675, 7.486)
**Total**	**1,589**	**680**	**0.4279421**	

a Pre- and post-*mimax* refer to the numbers of RAST annotations in each subsystem before and after *mimax* is run, respectively. Significant outliers are in bold.

b CI, confidence interval.

### Extending *mimax* to phage host prediction.

The preceding analysis indicates that *mimax* can identify genes with greater signatures of host specificity. We next ask whether this approach can be applied to the problem of virus host range prediction. We considered the simplest criterion for host prediction: a phage’s predicted host is the host with the greatest representation among homologs of the *mimax*-reduced genes within the phage’s genome. This criterion results in correct host assignment in 778 (85.2%) cases. This prediction accuracy is similar to that obtained by more sophisticated methods of host prediction that rely on comparison of k-mer frequencies between host and virus genomes (35–37).

**FIG 6:**
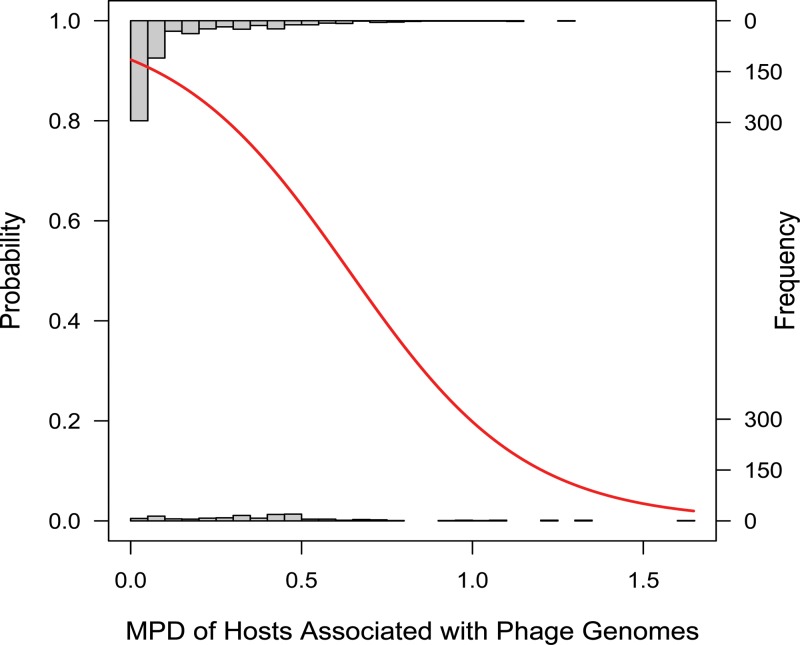
The probability that the most common host associated with the genes in a genome is the annotated host is negatively correlated with the diversity of hosts associated with these genes, as measured by MPD. Predictions are always correct when the MPD is 0 but incorrect in rare cases of low nonzero MPDs. (These cases account for the short bar that includes 0 on the *x* axis.) The distribution of MPDs for accurate predictions is shown as an inverted histogram along the top of the graph, whereas the MPD distribution for incorrect predictions is shown along the *x* axis. The red curve shows the probability distribution inferred by logistic regression (McFadden’s *R*^2^ = 0.169, *P* < 10^−16^).

Prediction accuracy varied across host genera, and incorrect host predictions tended to predict that phages infect closely related hosts (Table 2). Accuracy was negatively correlated with the MPD of hosts associated with phage genomes when restricted to the genes remaining after *mimax* (Fig. 6). Logistic regression confirms that it is easier to make accurate host predictions when the genes in a genome are associated with a narrow set of hosts. In fact, when only one host is associated with a genome (MPD = 0), the prediction is always correct. At the same time, it will be difficult to predict the host for more mosaic phage genomes, where genes are found across other phages infecting a wider variety of bacteria.

**TABLE 2:** Host accuracy varies with genus and sampling

Host genus	Accuracy	Most common incorrect prediction	No. of phages infecting host
*Chlamydia*	1.00	NA^a^	4
*Lactococcus*	1.00	NA	36
*Mycobacterium*	0.99	*Lactococcus*	226
*Bacillus*	0.97	*Chlamydia*	66
*Streptococcus*	0.95	*Bacillus*	38
*Escherichia*	0.91	*Salmonella*	138
*Prochlorococcus*	0.91	*Synechococcus*	21
*Staphylococcus*	0.90	*Bacillus*	87
*Pseudomonas*	0.85	*Escherichia*	85
*Burkholderia*	0.83	*Pseudomonas*	30
*Salmonella*	0.80	*Escherichia*	56
*Vibrio*	0.69	*Escherichia*	51
*Clostridium*	0.67	*Streptococcus*	21
*Acinetobacter*	0.58	*Escherichia*	12
*Shigella*	0.27	*Escherichia*	11
*Yersinia*	0.27	*Escherichia*	11
*Anabaena*	0.00	*Escherichia*	1
*Microcystis*	0.00	*Escherichia*	1
*Chlamydophila*	0.00	*Chlamydia*	1
*Synechococcus*	0.00	*Prochlorococcus*	15
*Bdellovibrio*	0.00	*Escherichia*	2

a NA, not applicable.

We also assessed this approach by using phages excluded from the original gene network. We chose 500 phage genomes at random from the new genomes published since we obtained our original data set. Of these, 185 were annotated as infecting host genera already included in the network. The genes in these phages were assigned using blastp to the *mimax*-reduced set of MCL clusters. Fifty-two of these phages shared no genes in the *mimax* set with any phages in our original data set. For the remaining 133 phages, our procedure predicted the host genus 67.7% of the time (see Table S1). This accuracy is lower than that found by other methods (35, 37) and is reduced further if one considers that many genomes could not be considered because they were outside the original network and host set.

10.1128/mBio.01870-17.7 TABLE S1  Summary of host prediction accuracy for phages outside the network. Download TABLE S1, DOCX file, 0.01 MB. Copyright © 2018 Shapiro and Putonti. 2018 Shapiro and Putonti This content is distributed under the terms of the Creative Commons Attribution 4.0 International license.

## DISCUSSION

In this work, we have shown that gene level networks offer both a high-resolution view of viral genetic diversity and a means to connect specific groups of genes to broad patterns in viral ecology and evolution. This network perspective also provides insights into the current and past ecology of phages. While many phages have no genes in common, in most cases, there exists a set of possible paths that can connect each of their genes in relatively few steps. In fact, the average shortest path distance between any two genes is only 3.2 edges. This means that two phages that appear unrelated on the basis of sequence similarity can still share an evolutionary history of gene exchange and loss. Moreover, the average shortest path distance between the genes of two phages is correlated with the phylogenetic relatedness of their hosts. This continuous relationship can be interpreted in two non-mutually exclusive ways: (i) phages whose genes are closer in the network (or that have more shared edges) are more likely to infect the same hosts, and (ii) phages with more similar hosts are likelier to be closer in the network. We have focused largely on the former, but the latter is equally meaningful. It serves as a reminder that for two phages from seemingly different lineages to share genes (or to each share genes with a third phage), they must have ancestors that infected the same host in the past.

It is important to note that while gene network topology is significantly correlated with host phylogenetic relatedness, it is not a perfect relationship, and host relatedness accounts for only about one-third of the variation in the average shortest path distance between phage genomes. Several factors might affect the strength of this correlation. Most notably, two phages that infect the same host will rarely have identical sets of genes. Thus, the amount of genetic variation among phages infecting the same host establishes a ceiling on how well host relatedness can correlate with gene network topology. This can be seen in both panels of Fig. 3 as the wide stretch of points when the host phylogenetic distance equals zero.

We also demonstrated how the gene network can inform virus host range predictions. As described above, accuracy is affected by the variation in gene content among phages infecting the same host, and we developed *mimax* to identify genes that reduce this source of noise. The majority of phage gene functions were not significantly affected, meaning that their presence or absence did not correspond to the mutual information between MCL clustering and host associations. One reason for this effect is that many genes with different functions are connected within a single MCL cluster. When removing a cluster improves the mutual information with host assignment, this will also reduce the number of representatives for other gene functions. How these genes covary among genomes will therefore affect their potential to affect mutual information. Further, many genes that affect virus host range, such as tail fiber genes, have significant phenotypic differences due to single point mutations (e.g., see reference 38). Their presence or absence among phage genomes will likely not help inform host range, but small sequence variation within individual nodes representing these genes will. Gene functions that are not significantly enriched by *mimax* should, therefore, not be viewed as unimportant, since *mimax* only considers patterns of presence and absence. Tail fiber genes are not identified by *mimax*, and this likely reflects the fact that small variations within these genes can have large phenotypic effects. Our methods currently do not incorporate this sequence level variation, and future work should account for this level of variation.

The accuracy of host predictions also varies significantly with both the sampling of phages on different hosts and the variation in gene exchange among viruses infecting different host genera. When a virus has few connections to the network, host prediction accuracy will be limited. Expanding the network to include more host genera and greater diversity of phages infecting each host will increase the potential to base hypotheses on network inference. In conjunction with new virus genomes from metagenomic data, wet lab characterization of environmental isolates on diverse hosts would also bolster the capacity of the network to inform broad questions in phage ecology and evolution.

One should also be careful when assessing the quality of negative predictions. While phage host range can be exceptionally specific, many phages infect multiple genera (39–41) and additional lab work is required to confirm that putatively incorrect predictions are not, in fact, false-negative results. In some cases, incorrect predictions may indicate host breadth rather than computational error, and future work should examine if measures like MPD can be extended to estimate the probability of infecting multiple host genera. Similarly, incorrect host predictions may signal the capacity of a virus to evolve to infect the predicted host.

Last, while our focus has been on virus host range, gene network analysis should be extensible to other aspects of viral ecology, including isolation source (e.g., freshwater, marine, soil, leaf, gut, hospital, etc.) and abiotic or biotic factors that vary across locations (e.g., temperature, pH, O_2_, nutrient concentrations, and available host diversity). These variables may correlate with distinct sets of genes, and tools like *mimax* can help identify these differences. Phages have a direct impact on the growth of their host bacteria, and knowing a phage’s ecological and evolutionary history is critical to understanding how that phage affects an ecosystem.

## MATERIALS AND METHODS

### Virus genomes.

All available phage RefSeq genomes were downloaded from NCBI in October 2014. These phages include members of the families *Myoviridae*, *Siphoviridae*, *Podoviridae*, *Microviridae*, *Inoviridae*, *Leviviridae*, *Cystoviridae*, and *Tectiviridae*, and unclassified phages. Five of the tailed phages were only annotated as *Caudovirales*. All new tailed phage genomes were downloaded from NCBI in October 2015 to supplement this list. Of this initial set of 1,328 genomes, 945 were annotated with a host in the GenBank metadata. Additional genomes have been published since these sequences were downloaded. We downloaded a random sample of 500 newer sequences to test the host prediction methods described below.

### Virus gene clustering.

Each virus genome was downloaded as a GenBank flat file and converted to FAA format, which consists of the individual protein sequence encoded by each gene in a genome. All virus FAA files were concatenated into a single FASTA file and clustered with USEARCH (26) by using the cluster_fast function with identity thresholds ranging from 20 to 95%. Clusters with three or more members were retained for network construction. While increasing the identity threshold results in more, smaller clusters, removal of singleton and doubleton clusters results in fewer clusters (and fewer included genomes) as this parameter is increased. Previous work on phage gene clustering found that 32.5% amino acid identity provided a suitable balance between the rate of finding new clusters and the percentage of singleton clusters (14). We found that 35% provided a similar balance for our data set (Fig. S6), yielding 32,897 protein clusters, of which 19,412 were singletons, 4,638 were doubletons, and 8,847 contained at least three members. These clusters contained genes from 913 of the 945 initial genomes.

10.1128/mBio.01870-17.6 FIG S6  Scatterplots showing that increasing the identity threshold in USEARCH increases both the total number of clusters found (a) and the frequency of singleton clusters (b). Download FIG S6, EPS file, 0.1 MB. Copyright © 2018 Shapiro and Putonti. 2018 Shapiro and Putonti This content is distributed under the terms of the Creative Commons Attribution 4.0 International license.

### Network construction.

Adjacency matrices and network edge lists were created in R (42). We built a genome-gene presence/absence matrix, *P*, in which each entry {*i*,*j*} was 1 if virus genome *i* contained a homolog found in gene cluster *j*. This matrix had dimension of 913 by 8,847 and is equivalent to the adjacency matrix for a bipartite network of phage genomes and genes. Adjacency matrices for the genome and gene level networks were then created as *A*_genome_ = sign(*P* × *P*^*T*^) and *A*_gene_ = sign(*P*^*T*^ × *P*), where *T* indicates the matrix transpose. The sign() function replaces all nonzero entries resulting from the original matrix products with a 1, converting the matrices from weighted to unweighted adjacency matrices. These matrices were then transformed into undirected graphs and corresponding edge lists using igraph (43). Thus, for the genome-level network, two genomes are considered connected if they share any genes, and two genes are connected in the gene-level network if they are ever found within the same genome.

### Network analysis.

Graphical clusters within the genome and gene networks were identified by using the Markov Cluster algorithm (MCL), as in MCL-edge (27, 44; also see http://micans.org/mcl) and OrthoMCL (45). MCL iteratively transforms an input adjacency matrix by inflating values to minimize a measure of chaos in the network. This results in the classification of nodes with similar patterns of connectedness into separate clusters. MCL methods have been used in prior work with genome-level networks to group phage genomes into clusters (12, 13, 16). We ran MCL on both genome- and gene-level networks with inflation parameters ranging from 1.2 to 6. We calculated the ICCC (16) to assess the level of cohesion within each set of MCL clusters. The R function findICCC() is available in the GitHub repository noted below.

### Estimating MPD of hosts associated with genes and genomes.

Many genes are found in phages infecting different hosts, and the distribution of these hosts was recorded in a vector for each gene. For example, if a gene has homologs in three phages that infect *Escherichia*, *Salmonella*, and *Yersinia*, then it would have a 1 in the corresponding position in the vector for each of these hosts and a 0 elsewhere. Each phage genome was then represented by the sum of the host association vectors for its genes.

We summarized the overall diversity of hosts associated with each gene or genome by using mean pairwise phylogenetic distance (MPD) (32). To calculate MPD, we first built a phylogeny for the bacterial host strains annotated as the host in the GenBank file for each phage. The tree was made using a set of conserved single-copy genes (as described in reference 46). Homologs were aligned with MUSCLE (47), and the final tree was inferred with FastTree (48) and visualized using iTOL (49). We then derived a genus-level tree by collapsing leaves with a shared genus to their common ancestor using the package ape in R (50). To collapse these leaves, we treated *Chlamydophila abortus* as a member of *Chlamydia*. *Burkholderia cepacia* was misplaced with *Mycobacterium* in the species tree and was excluded when the genus-level host phylogeny was built. The tree distance matrix was calculated using the function cophenetic() (also in ape). The host association vectors (see “Quantifying associations between graph clusters and phage hosts”) were then compiled into a matrix with rows corresponding to either genes or genomes and columns corresponding to hosts. This matrix and the tree distance matrix were then used as inputs for the function mpd() in the R package picante (51) with the option abundance.weighted set to TRUE.

MPD values were then compared to two measures of phage genome similarity based on network topology. The first, average shortest path distance, finds the average number of edges in the gene network separating each pair of genes in two phage genomes. All pairs of shortest path distances between genes were calculated using the igraph distances() function and stored in a symmetrical matrix where position {*i*,*j*} provides the distance between genes *i* and *j*. The average shortest path distance separating two phage genomes is then the average value for the submatrix where rows are restricted to genes from the first genome and columns are restricted to genes from the second genome. The second measure, network similarity, gives the proportion of edges shared by two nodes in a network. A pairwise similarity matrix was estimated analogously to the distances matrix described above using the igraph function similarity(). The average similarity between genes of two genomes was then calculated from this matrix as for the average shortest path distance. MPD was then regressed against these two measures (Fig. 3).

### Estimating mutual information.

Mutual information measures the extent to which two variables reveal each other’s states. We used this metric to estimate how closely MCL clusters in the genome and gene networks corresponded to the annotated host associated with each node in the network. The mutual information, *I*, between two random variables *X* and *Y* is defined by the equation

(1) $$I(X,Y)=\underset{x\in X,y\in Y}{\sum }p(x,y)\log\left(\frac{p(x,y)}{p(x)p(y)}\right)$$

where *x* and *y* are the values observed for the variables *X* and *Y*. By convention, individual values are set to 0 if *p*(*x*, *y*) = 0.

To estimate the mutual information between MCL clusters and host associations in each network, we first built a matrix, χ, of MCL cluster-host associations where rows corresponded to MCL clusters and columns corresponded to the hosts in our data set. For each entry {*i*,*j*} in χ, we summed the number of members of MCL cluster *i* that were annotated as infecting host *j*. For the gene network, we summed across all of the hosts associated with a node and then across all of the nodes within an MCL cluster to determine the host vector for each MCL cluster. Given χ, the rows and columns can be considered the *X* and *Y* variables for calculating the mutual information as in equation 1 above. We implemented this calculation with an R function [micalc() in the GitHub repository].

### Comparing gene annotations before and after *mimax*.

We annotated the full set of genes and the subset with the highest mutual information following *mimax* (described in Results) using RAST (33). We then identified significantly over- and underrepresented annotations following *mimax* by looking for outliers in a linear regression of the annotation frequencies after *mimax* against the frequencies before *mimax* (Fig. 5). The regression itself was highly significant (*R*^2^ = 0.93, *P* < 10^−16^), and outliers were those points lying above or below a 99.9% confidence interval around the line of best fit. This criterion is more stringent than the Bonferroni correction for multiple comparisons to compare each of the 32 points to their values predicted by the regression model (assuming a significance threshold of 0.05 for a single test).

### Predicting hosts for phages inside and outside the network.

The methods described above were also extended as a possible means for phage host prediction. Each phage included in the network was described in the calculation of MPD by a host association vector. The host for each phage was then predicted as the most frequent host among the genes (according to this vector), excluding contributions from the phage’s own genome to the result. When a phage was not a member of the original data set, we used blastp to align each of its genes with the centroid sequence for each node in the gene network. Hits were considered significant if the E value was below 10^−5^ and if the bit score was >50. Significant matches to genes contained in the *mimax*-reduced data set were then used to establish a host association vector for the phage and to predict the host as described above.

Logistic regression of prediction accuracy against the MPD of hosts associated with phage genomes was performed using the function glm() in R. McFadden’s *R*^2^ for the logit model was estimated using the function pR2() in the package pscl (52). The plot in Fig. 6 was made with a version of the function logi.hist.plot() from the package popbio (53) that was modified to allow for smaller bins.

### Network visualization.

Networks were visualized in Cytoscape3 (54) using the Prefuse Force-Directed Layout by importing the edge lists.

### Data and software availability.

Nucleotide sequence accession numbers, data, and code used in this work are available through figshare at https://figshare.com/s/cba533ddfd55e9cf75a8 and also through GitHub at https://github.com/coevoeco/GeneNet.
