# Supplementary figures and images for: Gene Co-occurrence Networks Reflect Bacteriophage Ecology and Evolution
Source: mBio. 2018 Mar 20;9(2):e01870-17. doi: 10.1128/mBio.01870-17 (PMC5874904; doi:10.1128/mBio.01870-17)

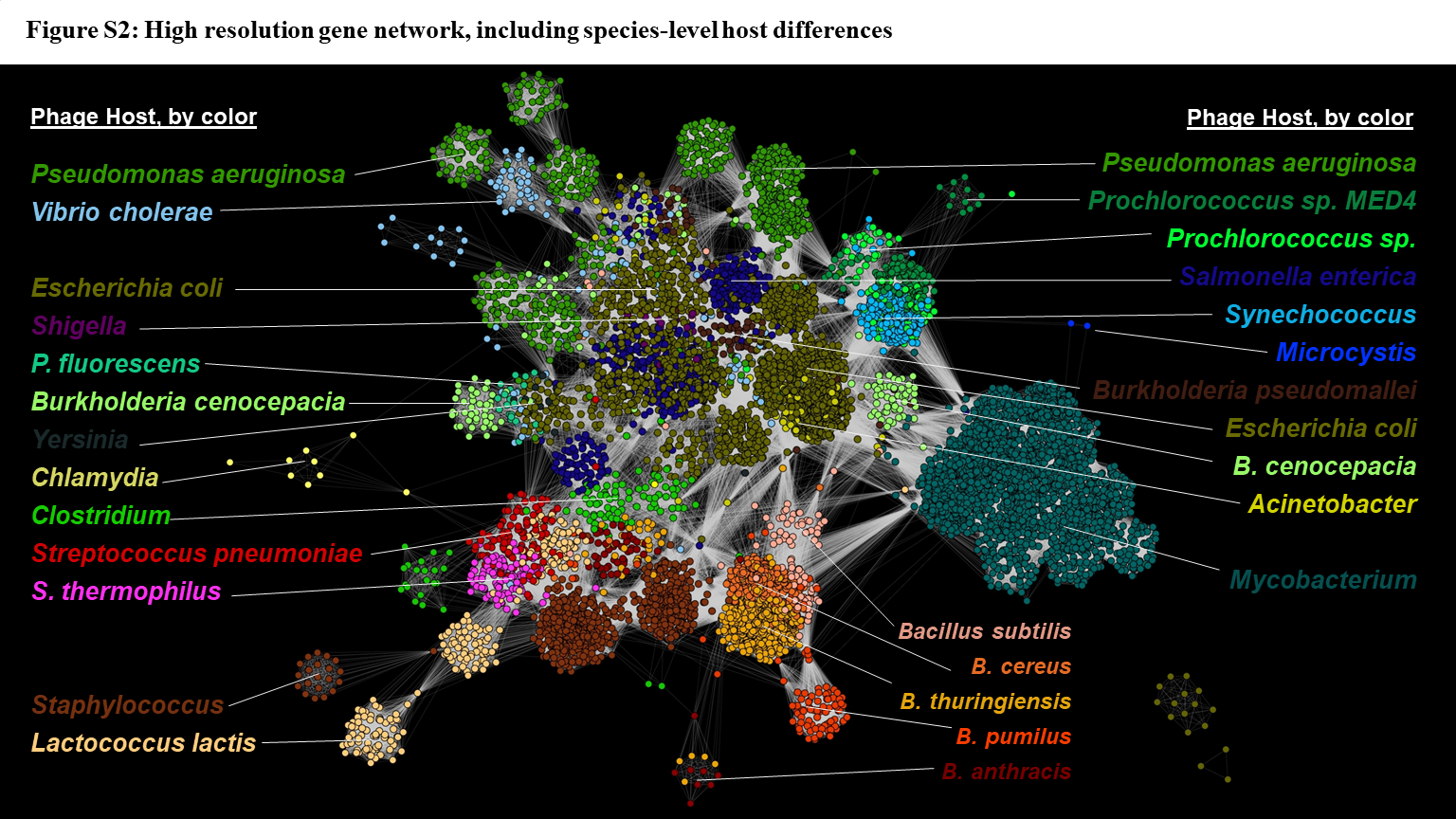

Supplement: FIG S2 [file mbo002183780sf2.tif]
