# Supplementary material for: Gene Co-occurrence Networks Reflect Bacteriophage Ecology and Evolution
Source: mBio. 2018 Mar 20;9(2):e01870-17. doi: 10.1128/mBio.01870-17 (PMC5874904; doi:10.1128/mBio.01870-17)
Supplement: TABLE S1 [file mbo002183780st1.docx]

**Supplemental Table S1: Host accuracy variation with novel phage sample**

| **Host Genus** | **Accuracy** | **Most common**  **incorrect prediction** | **# of phages**  **infecting host** |
| --- | --- | --- | --- |
| *Staphylococcus* | 1.00 | N/A | 5 |
| *Prochlorococcus* | 1.00 | N/A | 2 |
| *Mycobacterium* | 0.97 | *Pseudomonas* | 42 |
| *Escherichia* | 0.87 | *Salmonella* | 30 |
| *Lactococcus* | 0.83 | *Escherichia* | 12 |
| *Streptococcus* | 0.67 | *Escherichia* | 7 |
| *Pseudomonas* | 0.65 | *Escherichia* | 18 |
| *Clostridium* | 0.50 | *Bacillus* | 6 |
| *Acinetobacter* | 0.50 | *Escherichia* | 6 |
| *Bacillus* | 0.42 | *Escherichia* | 23 |
| *Salmonella* | 0.40 | *Escherichia* | 11 |
| *Synechococcus* | 0.00 | *Prochlorococcus* | 10 |
| *Shigella* | 0.00 | *Escherichia* | 5 |
| *Vibrio* | 0.00 | *Escherichia* | 4 |
| *Microcystis* | 0.00 | *Escherichia* | 1 |
